# Staple line lockstitch reinforcement decreases clinically relevant pancreatic fistula following distal pancreatectomy: Results of a propensity score matched retrospective analysis

**DOI:** 10.3389/fonc.2022.999002

**Published:** 2022-10-21

**Authors:** Feng Tian, Ming-jie Luo, Meng-qing Sun, Jun Lu, Bo-wen Huang, Jun-chao Guo

**Affiliations:** ^1^ Department of General Surgery, State Key Laboratory of Complex Severe and Rare Diseases, Peking Union Medical College Hospital, Chinese Academy of Medical Sciences and Peking Union Medical College, Beijing, China; ^2^ Chinese Academy of Medical Sciences and Peking Union Medical College, Beijing, China; ^3^ State Key Laboratory of Ophthalmology, Zhongshan Ophthalmic Center, Sun Yat-sen University, Guangzhou, Guangdong, China

**Keywords:** CR-POPF, distal pancreatectomy, staple line, lockstitch reinforcement, pancreatic fistula

## Abstract

**Background:**

Postoperative pancreatic fistula (POPF) remains the primary complication of distal pancreatectomies. We aimed to review whether staple line reinforcement with continuous lockstitches would lead to decreased grade B and C pancreatic fistula in patients undergoing distal pancreatectomy.

**Methods:**

This retrospective study enrolled consecutive patients scheduled to undergo distal pancreatectomy at a large tertiary hospital. A comparison was conducted between lockstitch reinforcement and non-reinforcement for remnant closure during distal pancreatectomies from August 2016 to February 2021. Propensity score matching was applied to balance the two groups with covariates including abdominal and back pain, diabetes mellitus, and estimated blood loss. The primary outcome was POPF rate.

**Results:**

A total of 153 patients were enrolled in the study (89 lockstitch reinforcements, 64 non-reinforcements), of whom 128 patients (64 per group) were analyzed after propensity score matching (1:1). The total POPF rate was 21.9%. POPF was identified in 12.5% (8/64) of the patients who underwent resection with lockstitch reinforcement and 31.2% (20/64) of the patients without reinforcement (odds ratio 0.314, 95% confidence interval 0.130-0.760, P=0.010). No deaths occurred in either group. Neither the major complication rate nor the length of hospital stay after surgery differed between the groups.

**Conclusions:**

Compared with the use of stapler alone, staple line lockstitch reinforcement for remnant closure during distal pancreatectomy could reduce the POPF rate. Further multicenter randomized clinical trials are required to confirm these results.

## Introduction

Distal pancreatectomy (DP) is the standard surgical procedure for benign, premalignant, or malignant pancreatic tumors located in the body and tail of the pancreas (1). According to the published literature, post-DP morbidity varies from 5–64% in different centers (2–4). Postoperative pancreatic fistula (POPF) remains the major complication after DP and can potentially cause further complications, such as abdominal fluid collection, severe intra-abdominal infection and hemorrhage. Preventing POPF *via* effective pancreatic remnant closure remains challenging, and no consensus on the optimal surgical technique has been established (1, 5–8).

Surgical staples have been widely applied for remnant closure because of their convenience and the mature laparoscopic DP technique used. However, the DISPACT trial demonstrated non-superior results with similar POPF rates in stapler versus scalpel resection followed by hand-sewn closure of the pancreatic remnant (9). Various surgical techniques for staple line reinforcement have been reported to prevent POPF, including reinforced staples, stump coverage with autologous tissue, absorbable or nonabsorbable mesh, and biological glue. However, when compared with stapler or hand-sewn closure, most of the methods showed no convincing benefit in terms of POPF (10–16).

The effective closure of pancreatic remnants of irregular thickness is crucial for fistula prevention. This study reviewed a propensity score matched cohort of patients who underwent DPs with or without splenectomy and compared the efficacy of staple plus lockstitch reinforcement versus non-reinforcement (staples only) on the POPF rate.

## Methods

### Study design and patient enrolment

This retrospective study included patients scheduled to undergo DPs between August 2016 and February 2021 at the Peking Union Medical College Hospital. Preoperative candidate diagnoses included pancreatic malignancies, pancreatic neuroendocrine tumors, pancreatic cystic neoplasms, chronic pancreatitis, and pancreatic pseudocysts. All patients were identified from a medical record-based database at the authors’ institution. A single experienced surgeon, who had performed more than 400 pancreatectomies, performed all the surgeries. This study was approved by the institutional ethics committee (approval number: S-K1937). Written informed consent was obtained from all participants.

### Inclusion and exclusion criteria

The inclusion criteria were as follows: patients of both sexes scheduled to undergo DPs with or without splenectomy for either benign or malignant neoplasms; preoperative diagnoses of serous or mucinous cystic adenoma, solid pseudopapillary tumor, neuroendocrine tumor, intraductal papillary mucinous neoplasm, pseudocyst, or distal pancreatic malignancies; use of a stapler when closing the pancreatic remnant; and willingness to provide informed consent. The exclusion criteria were as follows: history of major upper abdominal surgeries; history of splenectomy, gastrectomy, liver resection, or duodenal or pancreatic resection (not including laparoscopic cystectomy); patients with pancreatic trauma; patients who underwent other procedures except DPs, such as pancreaticoduodenectomy, segmental pancreatic resection, enucleation, or exploration; no use of a stapler for remnant closure; and patients with pneumoperitoneum or severe cardiopulmonary contraindications who were unfit for surgery.

### Grouping and surgical technique standardization

**Figure 1:**
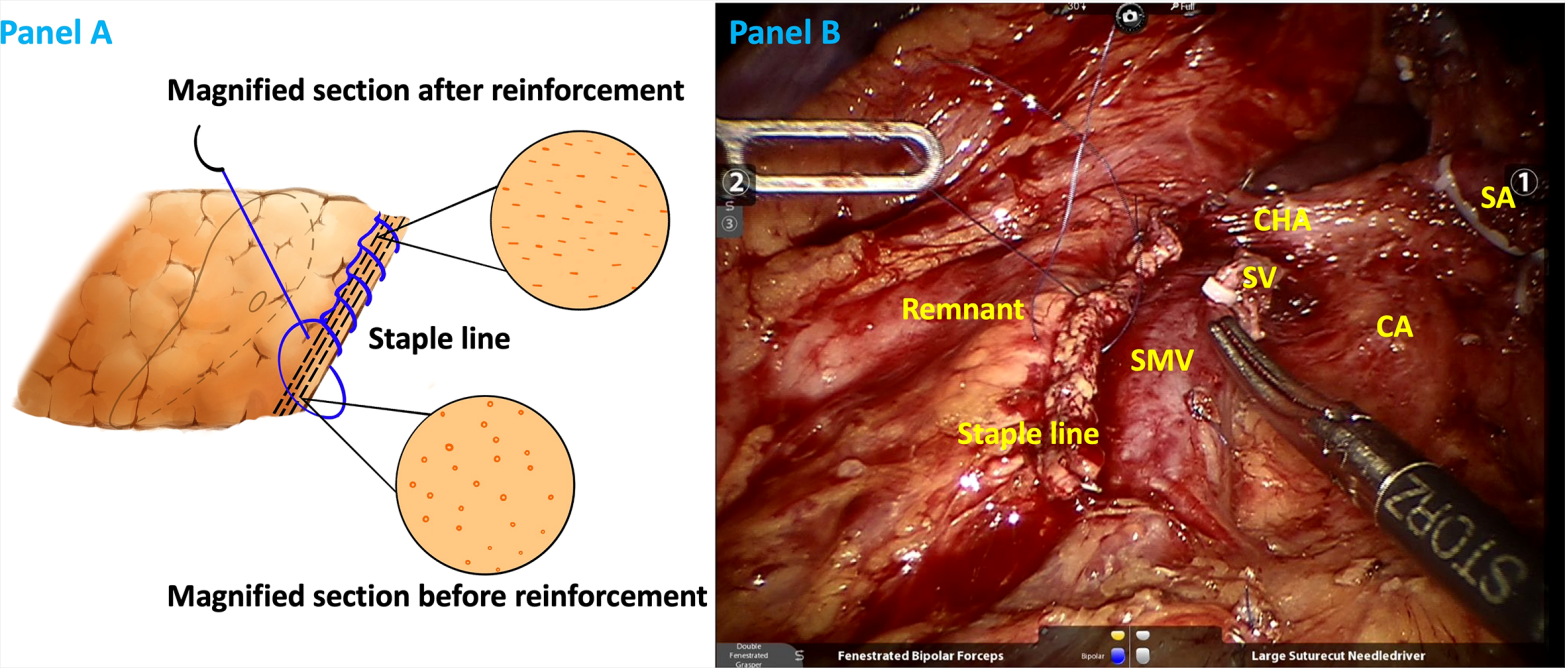
Illustrations of staple line reinforcement with continuous lock stitches in schematic **(A)** and realistic drawings **(B)**. SA splenic artery (ligated), CA celiac axis, CHA common hepatic artery, SV splenic vein (ligated), SMV superior mesentery vein.

The included patients were enrolled in two groups according to the closure style of the pancreatic remnant: lockstitch reinforcement of the staple line and no reinforcement (staple only). Initially, lockstitch reinforcement was mainly performed when a staple fire was less than optimal, such as fracture of the pancreatic tissue or remnant bleeding (after 2019, we performed lockstitch reinforcement in majority of the cases, regardless of staple line performance). The study group in which continuous lockstitches were placed along the staple line after transecting the pancreas (**Figure 1**) was set as the reinforcement group. Control group, i.e., non-reinforcement group, did not receive additional reinforcement after transecting the pancreas with a stapler.

Regarding the surgical approach, we considered heterogeneous tumor location and its relationship to the left wall of portal vein (PV) and the roots of splenic vessels. When the lesion was located near PV or even invaded the roots of splenic vessels, named “shoulder” pancreatic tumor in our previously published article, we preferred retrograde artery first approach pancreatosplenectomy (17). When the lesion was located far from PV and there was enough space to ligate the splenic vessels, we preferred radical antegrade modular pancreaticosplenectomy (RAMPS) (18). For key surgical steps during minimally invasive RAMPS, the authors’ team transected the pancreas before ligating the splenic vessels unless the splenic artery was easy to expose. In that case, the splenic artery was ligated first. After transecting the pancreas, we ligated the splenic vein and then the splenic artery considering the foot-to-head view under laparoscopy. Normally, we resected the pancreas at the neck if the lesion was located at the body and near the PV. If the lesion was far from PV or near the tail of the pancreas, we would transect the pancreas approximately 2 cm right to the lesion to leave normal parenchyma intact as much as possible.

A 60-mm stapler with different heights (Powered Echelon Flex stapler from Johnson & Johnson Medical Company, USA) was used for pancreatic transection. The frequently chosen stapler height was 3.6 mm, whereas a 2.6 mm height was chosen when the targeted parenchyma was particularly thin. In the study group, 5-0 Prolene (Ethicon, Somerville, NJ, USA) was used to perform lockstitches, with a needle gauge of approximately 5 mm, and was pulled tightly according to various thicknesses and firmness of the pancreas.

Two intra-abdominal drainages were routinely placed in all cases (one near the pancreatic remnant and the other in the spleen nest if splenectomy was performed simultaneously). Prophylactic somatostatin analogs, such as octreotide (Merck Serono, Aubonne, Switzerland) were used 1–3 days postoperatively according to the intraoperative performance and amylase levels.

Drain amylase levels were tested on postoperative day(s) 1, 3, 5, 7, and so on. The criteria for drainage removal were strict at the authors’ institution. Generally, the drainage was removed when the amylase levels were less than three times the upper normal institutional limit and the patient was asymptomatic. For patients with elevated amylase levels less than 5000 U/L and no intra-abdominal fluid collection, we removed the drainage on postoperative days 5-7. If the amylase levels were higher than 5000 U/L, we initiated the removal process when the drain volume was less than 10 mL per day and lasted for at least 3 days. In detail, we retracted the drainage gradually (3–5 cm at a time) until removal.

The patients met the discharge criteria when they resumed activity and autonomous eating, were afebrile, and did not need fluid transfusions. Whether the drain tube had been removed was not a determinant of discharge. Thus, some patients were discharged with the drainage still in place, which would be removed at the surgical clinic once the patient met the aforementioned criteria.

### Outcomes and data collection

The primary outcome measure was POPF, defined and identified according to the 2016 version of the International Study Group on Pancreatic Surgery (ISGPS) classification and grading of POPF (19). POPF is defined as the drain output of any measurable fluid volume with amylase levels greater than three times the upper limit of the institutional normal serum amylase level (115 U/L at the authors’ institution) associated with one or more clinical conditions related directly to the POPF. For the classification of POPF, different physicians’ interpretations may deviate from the ISGPS definition, causing potential bias. Therefore, two investigators independently performed POPF classification (TF and LMJ). If an inconsistency occurred, a senior professor reviewed this and made a judgment (GJC).

The second outcome included surgical variables (parenchymal firmness, operative time, lockstitch reinforcement time, estimated blood loss [EBL], blood transfusion rate, and conversion rate], short-term postoperative complication rate within 90 days, and pathological results [final pathologic diagnosis, margin status, and the number of harvested lymph nodes]). The postoperative length of stay (LOS) was also recorded. Due to the retrospective nature of the study, we did not collect data on the duration of performing lockstitch reinforcements; however, data were collected from several random samples of surgical videos. The R0 resection rate was defined as a tumor within 1 mm of the specimen margin (20). Definitions of postoperative complications, such as delayed gastric emptying (DGE) (21), post-pancreatectomy hemorrhage (22), and abdominal infection (23, 24), have been reported previously. The Clavien–Dindo classification was adopted to describe the severity of postoperative complications (25), with grade III or higher considered as a major complications.

In addition to the above variables, demographic data including age, sex, body mass index (BMI), American Society of Anesthesiologists (ASA) status, symptoms, medical history, carbohydrate antigen 19-9 level, and maximum tumor size measured on preoperative computed tomography (CT) scans were also collected using a standardized form.

### Postoperative follow-up

The first follow-up was arranged 30 days postoperatively and the second 90 days postoperatively at the outpatient clinic or by phone if the patient could not attend the clinic. Medical history, physical examination, and laboratory tests were performed routinely. Non-enhanced or contrast-enhanced CT scan was performed accordingly. For patients with the surgical drainage, the surgical drains were removed in the clinic when the drain volume was less than 10 mL per day and lasted for 3 days.

### Propensity-score matching

Mann–Whitney U tests and χ^2^ tests were conducted for patient demographics and clinicopathological characteristics. Significant between-group differences in the symptoms, rates of diabetes mellitus, and intraoperative blood loss were identified, which might potentially affect the risk of POPF. Characteristics which possibly contribute to fistula such as pancreatic firmness, BMI, or sex did not differ between the groups (**Supplementary Tables**). Subsequently, we conducted propensity score matching (PSM) (staple plus reinforcement vs. non-reinforcement in a 1:1 match) to balance the two groups. The covariates included abdominal and back pain, diabetes mellitus, and EBL. The pancreatic remnant closure technique was used as the dependent variable for the PSM.

### Statistical analysis

PSM and statistical analyses were performed using R (R Core Team, 2018) and SPSS^®^ version 20.0 (IBM, Armonk, New York, USA). Continuous variables are described as medians (range) after testing for normality. Categorical variables are presented as frequencies and percentages. Student’s t-test or Mann–Whitney U test was used for continuous variables, and χ^2^ and Fisher’s exact tests were applied for categorical variables. P-values were considered significant at P <0.05.

## Results

**Figure 2:**
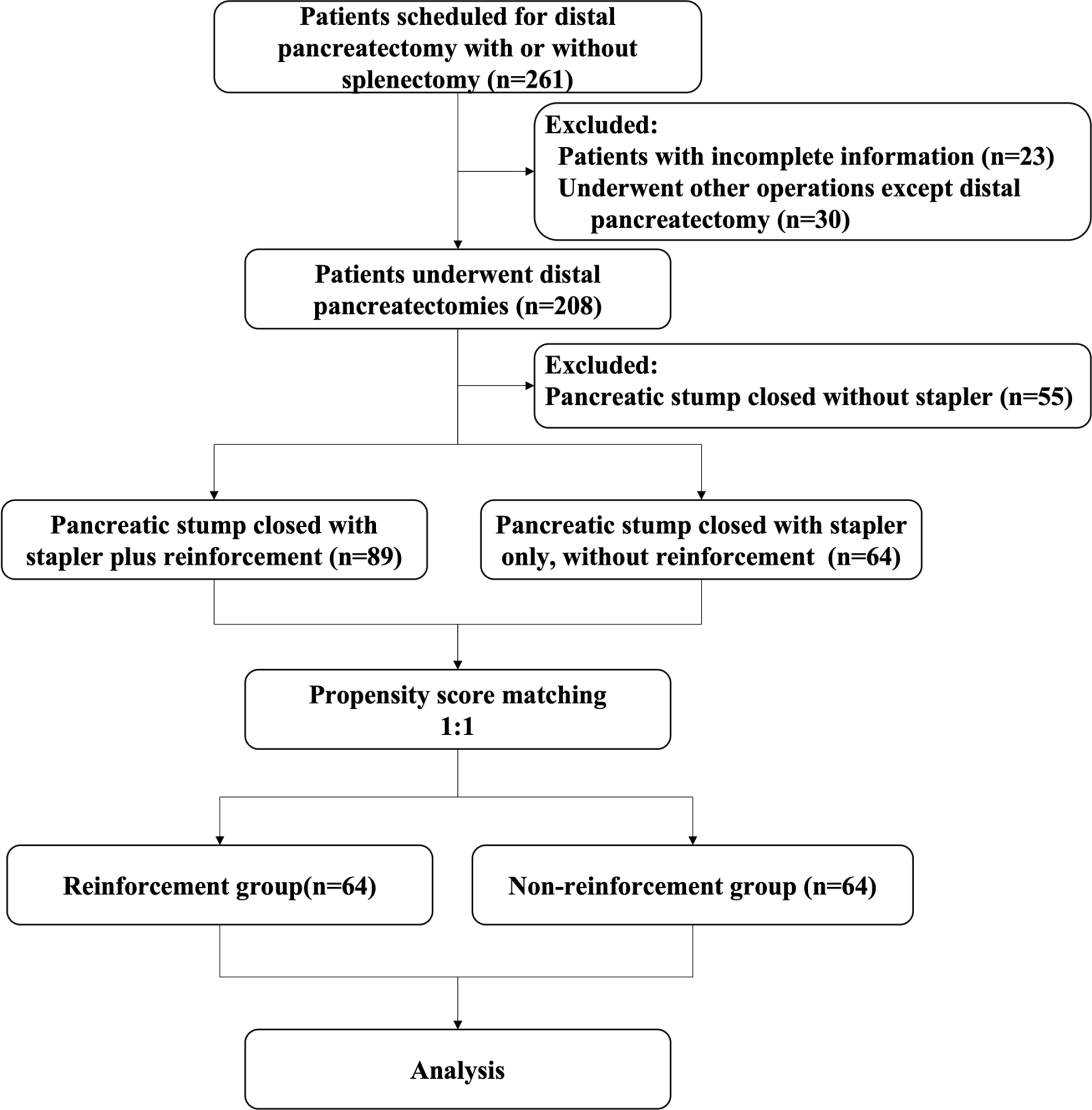
Study profile.

A total of 261 patients with distal pancreatic lesions were eligible for enrolment between the study intervals. Of these, 53 patients had to be excluded because procedures other than DP were performed (n=30), or the required data were incomplete (n=23). Thus, the intention-to-treat population consisted of 208 patients, of whom 55 did not use a stapler for remnant closure were excluded. A total of 153 patients were enrolled according to the inclusion criteria before matching, of whom 89 adopted staplers plus lockstitch reinforcement and 64 used only staplers without reinforcement. After PSM, a balanced cohort was created with 64 patients in each of the study and control groups (**Figure 2**).

The top five pathologies among the 208 patients who underwent DP were pancreatic adenocarcinomas (32.7%), solid pseudopapillary tumors (17.8%), serous cystic adenomas (11.5%), neuroendocrine tumors (10.1%), and mucinous cystic adenomas (10.1%). There were also a few rare pathological types, including adenosquamous carcinoma (n=2), acinar cell carcinoma (n=1), metastatic lesions from breast cancer (n=1), liposarcoma (n=2), leiomyoma (n=1), spindle cell sarcoma (n=1), tubular villous adenoma (n=1), and hemangioma (n=1; **Table 1**)

**Table 1:** Pathological array of 208 distal pancreatectomies.

Pathology	n(%)
Pancreatic ductal adenocarcinoma	68 (32.7)
Solid pseudopapillary tumor	37 (17.8)
Serous cystic adenoma	24 (11.5)
Neuroendocrine tumor	21 (10.1)
Mucinous cystic adenoma	21 (10.1)
Intraductal papillary mucinous neoplasm	14 (6.7)
Chronic pancreatitis	13 (6.2)
Other rare pathologic types*	10 (4.8)

*Including: adenosquamous carcinoma (n=2); alveolar cell carcinoma (n=1); metastatic cancer from breast cancer (n=1); liposarcoma (n=2); tubular villous adenoma (n=1); liomyoma (n=1); spindle cell sarcoma (n=1); hemangioma (n=1).

The study population comprised 48 men (37.5%) and 80 women (62.5%). There were no differences in the baseline data (age, sex, or BMI), clinical features (symptoms, accompanying medical histories, ASA status, tumor marker deviation), or radiological variables (tumor size, the relationship between the tumor and major vessels such as the portal vein-superior mesentery vein [PV-SMV] axis, and roots of splenic vessels) (**Table 2**).

**Table 2:** Comparison of baseline and clinicopathological characteristics of patients between the groups.

	Non-reinforcement group (n=64)	Reinforcement group (n=64)	*P* value^§^
Age, years ^*^	54.5 (13-81)	55.5 (17-79)	0.618^#^
Sex			0.273
Male	21 (32.8)	27 (42.2)	
Female	43 (67.2)	37 (57.8)	
Body mass index (kg/m^2^) ^*^	23.6 (15.6-32.7)	23.2 (15.4-33.6)	0.941^#^
Abdominal/back pain	31 (48.4)	26 (40.6)	0.374
Weight loss	17 (26.6)	25 (39.1)	0.132
Hypertension in medical history	14 (21.9)	18 (28.1)	0.414
Diabetes mellitus	8 (12.5)	7 (10.9)	0.783
Coronary heart disease	2 (3.1)	4 (6.2)	0.680^¶^
Cerebrovascular disease	2 (3.1)	3 (4.7)	1.000^¶^
Hyperlipemia	10 (15.6)	9 (14.1)	0.804
Maximum tumor size (cm) ^*^	5 (1.2-12)	4.8 (1-23)	0.960^#^
Preoperative pancreatic portal hypertension	11 (17.2)	13 (20.3)	0.651
PV/SMV axis invasion on imaging	10 (15.6)	15 (23.4)	0.265
Splenic artery invasion on imaging	13 (20.3)	12 (18.8)	0.824
Splenic vein invasion on imaging	9 (14.1)	12 (18.8)	0.474
Elevated CA19-9	18 (28.1)	20 (31.2)	0.699
Elevated CEA	11 (17.2)	11 (17.2)	1.000
Preoperative albumin (g/L) ^*^	43 (36-54)	44 (30-51)	0.521
Preoperative hemoglobin (g/dL) ^*^	133 (83-169)	136.5 (85-178)	0.333^#^
ASA Classification			0.571
Grade I or II	58 (90.6)	56 (87.5)	
Grade ≥III	6 (9.4)	8 (12.5)	
Pathology			0.461
PDAC	21 (32.8)	25 (39.1)	
Non-PDAC	43 (67.2)	39 (60.9)	

Values in parentheses are percentages unless indicated otherwise; *values are median (range). ASA, American Society of Anesthesiologists. PDAC, pancreatic ductal adenocarcinoma. §χ^2^ test, except ¶ Fisher’s exact test and #Mann–Whitney U test.

A total of 92.2% (118/128) of the DPs were completed *via* minimally invasive approaches, of which 6.3% (8/128) were converted to open surgeries for severe adhesion or uncontrollable hemorrhage. The minimally invasive surgery and conversion rates did not differ significantly between the two groups. Based on five random samples of surgical videos, the mean duration of performing lockstitch reinforcements was 521 ± 146.1 s (493, 614, 463, 327, 708 s, respectively). As shown in **Table 3**, the overall POPF rate was 21.9% (28/128), with rates of 12.5 and 31.2% in the reinforcement and non-reinforcement groups, respectively (P=0.010). Among the 28 patients with grade B POPF, 27 needed persistent drainage>21days (delayed removal of the surgical drainage), whereas no surgical, endoscopic, or radiological intervention were required. Only one patient needed percutaneous puncture due to intra-abdominal fluid and fever. No grade C fistula was observed.

**Table 3:** Comparison of rate of postoperative pancreatic fistula in staple line reinforcement and non-reinforcement groups .

	Non-reinforcement group (n=64)	Reinforcement group (n=64)	*P* value^§^
No leakage	7 (10.9)	10 (15.6)	0.601
Biochemical leak	37 (57.8)	46 (71.9)	0.138
POPF			0.010^*^
Grade B	20 (31.2)	8 (12.5)	
Grade C	0 (0)	0 (0)	

Values in parentheses are percentages. POPF, postoperative pancreatic fistula. §χ^2^ test, except *Fisher’s exact test.

The 90-day all-cause mortality rate was zero in both groups. The rates of spleen preservation and concomitant PV-SMV wall resection did not show any differences between the groups. Both groups were similar regarding parenchymal firmness, intraoperative median EBL, transfusion rate, and operative time. The duration of drainage tended to be shorter in the reinforcement group than in the non-reinforcement group (8 vs. 10 days, respectively; P=0.066). Major postoperative complications and LOS were similar between the two groups. There were two grade IIIa complications in the non-reinforcement group, including one case of DGE requiring a gastric tube reinsertion and one case of peripancreatic fluid accumulation with fever requiring reintervention, although pathogen cultures were all negative. In addition, there were nine grade II complications in both groups: one abdominal infection (*Enterococcus faecalis*), one blood infection (*Brucella*), and two intestinal infections (one *Candida albicans* and one *Clostridium difficile*), which were treated with antibiotics; three chylous leakages treated *via* fasting; and two patients with transient hemoglobin decline treated conservatively (**Table 4**).

**Table 4:** Comparison of safety and efficiency-related outcomes between the two groups.

	Non-reinforcement group (n=64)	Reinforcement group (n=64)	*P* value^§^
Surgical approach			0.510^¶^
Open	4 (6.2)	6 (9.4)	
Laparoscopic or robotic	60 (93.8)	58 (90.6)	
Conversion to open surgery	4 (6.3)	4 (6.3)	1.000^¶^
Parenchyma firmness			0.466
Soft	56 (87.5)	52 (81.3)	
Hard	8 (12.5)	12 (18.7)	
Operative time (min) ^*^	200 (100-460)	200 (110-440)	0.834^#^
Spleen preservation	12 (18.8)	12 (18.8)	1
Concomitant PV/SMV wall resection	8 (12.5)	8 (12.5)	1
Estimated blood loss (ml) ^*^	100 (20-1000)	125 (20-1000)	0.712^#^
Transfusion	6 (9.4)	8 (12.5)	0.571
Duration of drainage (days) ^*^	10 (6-60)	8 (3-60)	0.066^#^
Postoperative LOS (days) ^*^	9 (6-25)	10 (6-26)	0.378^#^
Clavien-Dindo classification			0.528^¶^
Grade I or II	46 (71.9)	28 (43.8)	
Grade IIIa^†^	2 (3.1)	0 (0)
Grade IIIb	0 (0)	0 (0)
Grade IV or V	0 (0)	0 (0)
90-day mortality	0 (0)	0 (0)	1

Values in parentheses are percentages unless indicated otherwise; *values are median (range). PV, portal vein. SMV, superior mesentery vein. LOS, length of stay. † Including one delayed gastric emptying and one peri-pancreatic fluid accumulation with fever needing reintervention. § χ2 test, except ¶ Fisher’s exact test and # Mann–Whitney U test.

## Discussion

The major finding of this study was that reinforcement of the staple line with continuous lockstitches resulted in a significantly decreased POPF rate compared with its nonreinforcement counterpart for DP (12.5 vs. 31.2%, P=0.010). Meanwhile, lockstitch reinforcement did not lead to differences in the major postoperative complication rate or patient recovery. Both remnant closure strategies were equally safe for DP.

This study presented a total POPF rate of 21.9% (28/128), which is similar to the 23% benchmark POPF rate reported in a study involving 3,016 patients from 24 randomized controlled trials undergoing DP (6). Risk scores for predicting POPF would promote preventive and mitigation strategies. Several studies have identified risk factors related to POPF occurrence after DP. Based on a retrospective study involving 2026 patients, Ecker et al. reported that age <60 years, obesity, hypoalbuminemia, absence of epidural anesthesia, nonmalignant pathology, concomitant splenectomy, and vascular resection were independent risk factors of POPF. Unfortunately, most of the factors were not modifiable and the prediction model showed unsatisfactory discrimination (1). Recently, Bonsdorff et al. and Pastena et al. developed and validated new risk scores for POPF after DP, introducing crucial risk factors, including the pancreatic thickness at the transection, the diameter of the pancreatic duct, diabetes, and the level of transection (neck or body-tail) (7, 8).

Effective closure of pancreatic remnants of irregular thickness is crucial for fistula prevention. The pancreatic parenchyma, particularly the soft and thick parenchyma, may be too fragile to retain the staples. The stapler may only tear the pancreatic tissue, potentially increasing the risk of leakage. Moreover, mismatch between the irregular remnant thickness and the stapler’s height might cause invisible minor leaks (**Figure 1**). Zimmitti et al. reported pancreatic capsule disruption and staple line bleeding at a high occurrence rate of 39% and 50%, respectively, during DPs. Moreover, they concluded that pancreatic capsule disruption and staple line bleeding were factors associated with higher POPF rate (26). The thicker the pancreas at the pancreatic transection site, the higher the possibility of disruption. Initially, for heterogeneous remnants, we only used electrocoagulation and single stitch for pancreatic capsule disruption and staple line bleeding. Obviously, no improvement of POPF was observed. Around 2019, we applied continuous lockstitch reinforcement along the staple line and found it might decrease POPF. From then on, we added reinforcement as a routine step during DP and applied it in majority of the cases, regardless of the occurrence of disruption. The logic of the staple line lockstitch reinforcement technique is that the fine lockstitches could close tiny pancreatic ducts according to different gland characteristics and tighten the remnant to the largest extent, thus preventing potential leakage from the remnant. It systemically, not focally, enhances the staple line and decreases POPF rate as demonstrated in our results, which is a reverse proof of the effectiveness of the lockstitch reinforcement technique. Therefore, the inconsistency between results of Zimmitti et al.’s study and our study lie in that they described the situation, and we put forward an alternative solution for this situation.

In the past decade, several studies have shown a significant reduction in POPF using reinforced stapler for closure of the remnant (10, 27, 28). However, a recent randomized trial reported no difference in terms of POPF or overall postoperative complications after DP comparing reinforced stapler versus standard stapler (14). Therefore, the potential superiority of reinforced stapler has not been confirmed and controversy remains (5). Moreover, the expenses could have limited the wide use of reinforced stapler. In the present study, the POPF rate in the reinforcement group is similar to the rate in reinforced stapler group (12%) reported by Wennerblom et al., even though they did not consider reintervention and the rate of POPF was possibly underestimated (14).

The reduced POPF rate should have shortened the postoperative LOS and the drainage duration in the reinforcement group, but there was no difference in the LOS (median 9 vs. 10 days in the reinforcement group; P=0.378) or duration of drainage (median 10 vs. 8 days in the reinforcement group; P=0.066) between the two groups. This may be related to our conservative strategies for postoperative management, especially the aspect dealing with surgical drains. We believe that around postoperative day 7, there is a high-risk period of pancreatic fistula due to tissue edema, necrosis, and increased secretion of pancreatic juice following oral intake. Therefore, we were accustomed to retaining the surgical drain until around postoperative day 7, unless the amylase levels were very low. This perhaps narrowed the difference of LOS and drainage duration between the two groups.

Surgical drainages are commonly used to mitigate POPF. Likewise, no-drain strategy in selected cases after DP was reported to not be associated with increased POPF rate when compared with routine prophylactic abdominal drainage (29, 30). However, the selection bias limits the conclusion of studies and controversy still exists (31). Future evidence is required for identifying which subset of patients is suitable for no-drain strategy. Prophylactic abdominal drainage has been reported to be associated with a greater fistula rate but reduced POPF severity (1). Strict criteria for drainage removal may increase inconvenience for patients after discharge. However, longer drainage may lower the possibility of intra-abdominal fluid collection and reduce the need for punctures. Meanwhile, drainage in place keeps an existing and easier pathway for a possible percutaneous drain. Wennerblom et al. (14) and Diener et al. (9), both reported a remarkably high rate of patients with intra-abdominal fluid and abscess (17–19%), majority of whom needed subsequent radiological or surgical reintervention. In this study, only one patient with fluid accumulation required percutaneous reintervention after removal of the drainage, leading to a very low Clavien–Dindo grade III or higher complication rate in both groups, which benefits patients. Concern might be raised that delayed drain removal was related to an increased incidence of bacterial contamination. However, in the present study, rare retrograde infection was detected. The low rate of Clavien–Dindo grade III or higher complication (3.1% vs. 0 in the reinforcement group; P=0.528) might also affect the detection of differences between the two groups. Of those with grade B fistula, most patients had prolonged intra-abdominal drainage (over 21 days) due to high drain amylase levels but no clinical symptoms.

This study had several limitations. First, although we applied PSM to decrease selection bias, inherent bias still existed in this retrospective study. For example, neither remnant characteristics, such as parenchymal thickness, duct diameter at the transection site nor the staple height were recorded, which was a potential source of bias. Second, this study described experience from a single surgeon, and repeatability of the reinforcement technique might be an issue if widely adopted.

In conclusion, compared with staplers only, stapler line reinforcement with lockstitches for remnant closure during DP could reduce the POPF rate. Randomized controlled trials are needed to validate the results of our study before generalizing the reinforcement technique. The quality of the reinforcement lockstitches, transection level, and pancreatic duct and parenchyma thickness at the transection site should be considered in future randomized controlled trials.

## Data availability statement

The raw data supporting the conclusions of this article will be made available by the authors, without undue reservation.

## Ethics statement 

The studies involving human participants were reviewed and approved by PUMCH Institutional Review Board. The patients/participants provided their written informed consent to participate in this study.

## Author contributions

FT: study design; acquisition of data; analysis and interpretation of data; drafting the article and revising it critically for important intellectual content. M-JL: acquisition and disposal data; data analysis and interpretation of data; and manuscript revision. M-QS: picture drawing; make contributions to conception and design; reviewed and revised the manuscript. JL: acquisition of data; make contributions to conception and design; reviewed and revised the manuscript. B-WH: acquisition of data; make contributions to conception and design; reviewed and revised the manuscript. J-CG: operated all the cases; make contributions to conception and design; manuscript revision; give final approval of the version to be published. FT and M-JL contributed equally to this work and share first authorship. All authors contributed to the article and approved the submitted version.

## Funding

This work was funded by the National Natural Science Foundation of China (grant number: 81972324), CAMS Innovation Fund for Medical Sciences (grant number: 2021-I2M-1-002), Bethune Charitable Foundation (grant number: FW-HXKT2019013000198), and National High Level Hospital Clinical Research Funding. The funding bodies played no roles in the design or conduction of this study, had no access to the data or role in data collection, management, analysis, or interpretation, and had no role in preparation, review, or approval of the manuscript.

## Conflict of interest

The authors declare that the research was conducted in the absence of any commercial or financial relationships that could be construed as a potential conflict of interest.

## Publisher’s note

All claims expressed in this article are solely those of the authors and do not necessarily represent those of their affiliated organizations, or those of the publisher, the editors and the reviewers. Any product that may be evaluated in this article, or claim that may be made by its manufacturer, is not guaranteed or endorsed by the publisher.
