# Supplementary material for: Staple line lockstitch reinforcement decreases clinically relevant pancreatic fistula following distal pancreatectomy: Results of a propensity score matched retrospective analysis
Source: Front Oncol. 2022 Oct 21;12:999002. doi: 10.3389/fonc.2022.999002 (PMC9634739; doi:10.3389/fonc.2022.999002)
Supplement: Supplementary file 1 [file Table_1.docx]

**Table 2** Comparison of baseline and clinicopathological characteristics of patients between the groups before PSM

|  |  | Non-reinforcement group (n=64) | Reinforcement group (n=89) | *P* value^§^ |
| --- | --- | --- | --- | --- |
| Age, years ^*^ |  | 54.5 (13-81) | 57 (17-83) | 0.206^#^ |
| Sex |  |  |  | 0.131 |
| Male |  | 21 (32.8) | 40 (44.9) |  |
| Female |  | 43 (67.2) | 49 (55.1) |  |
| Body mass index (kg/m^2^) ^*^ |  | 23.6 (15.6-32.7) | 23.7 (15.4-33.6) | 0.810^#^ |
| Abdominal/back pain |  | 31 (48.4) | 30 (33.7) | 0.066 |
| Weight loss |  | 17 (26.6) | 35 (39.3) | 0.100 |
| Hypertension in medical history |  | 14 (21.9) | 27 (30.3) | 0.244 |
| Diabetes mellitus |  | 8 (12.5) | 23 (25.8) | 0.043 |
| Coronary heart disease |  | 2 (3.1) | 7 (7.9) | 0.305^¶^ |
| Cerebrovascular disease |  | 2 (3.1) | 4 (4.5) | 1.000^¶^ |
| Hyperlipemia |  | 10 (15.6) | 11 (12.4) | 0.563 |
| Maximum tumor size (cm) ^*^ |  | 5 (1.2-12) | 4.7 (1-23) | 0.839 |
| Preoperative pancreatic portal hypertension |  | 11 (17.2) | 17 (19.1) | 0.763 |
| PV/SMV axis invasion on imaging |  | 10 (15.6) | 20 (22.5) | 0.293 |
| Splenic artery invasion on imaging |  | 13 (20.3) | 14 (15.7) | 0.463 |
| Splenic vein invasion on imaging |  | 9 (14.1) | 15 (16.9) | 0.640 |
| Elevated CA19-9 |  | 18 (28.1) | 36 (40.4) | 0.116 |
| Elevated CEA |  | 11 (17.2) | 19 (21.3) | 0.523 |
| Preoperative albumin (g/L) ^*^ |  | 43 (36-54) | 44 (30-51) | 0.771 |
| Preoperative hemoglobin (g/dL) ^*^ |  | 133 (83-169) | 136 (65-178) | 0.261^#^ |
| ASA Classification |  |  |  | 0.711 |
| Grade I or II |  | 58 (90.6) | 79 (88.8) |  |
| Grade ≥III |  | 6 (9.4) | 10 (11.2) |  |
| Pathology |  |  |  | 0.215 |
| PDAC |  | 21 (32.8) | 38 (42.7) |  |
| Non-PDAC |  | 43 (67.2) | 51 (57.3) |  |

Values in parentheses are percentages unless indicated otherwise; *values are median (range). ASA, American Society of Anesthesiologists. PDAC, pancreatic ductal adenocarcinoma. §χ^2^ test, except ¶ Fisher’s exact test and #Mann–Whitney *U* test.

**Table 3** Comparison of rate of postoperative pancreatic fistula in staple line reinforcement and non-reinforcement groups before PSM

|  | Non-reinforcement group (n=64) | Reinforcement group (n=89) | P value§ |
| --- | --- | --- | --- |
| No leakage | 7 (10.9) | 15 (16.9) | 0.356 |
| Biochemical leak | 37 (57.8) | 63 (70.8) | 0.121 |
| POPF |  |  | 0.007^*^ |
| Grade B | 20 (31.2) | 11 (12.4) |  |
| Grade C | 0 (0) | 0 (0) |  |

Values in parentheses are percentages. POPF, postoperative pancreatic fistula. §Fisher’s exact test, except *χ^2^ test.

**Table 4** Comparison of safety and efficiency-related outcomes between the two groups before PSM

|  | Non-reinforcement group (n=64) | Reinforcement group (n=89) | | P value§ | |  |
| --- | --- | --- | --- | --- | --- | --- |
| Surgical approach | | |  | | 0.210^¶^ | |
| Open | 4 (6.2%) | 11 (12.4%) | |  | |  |
| Laparoscopic or robotic | 60 (93.8%) | 78 (87.6%) | |  | |  |
| Conversion to open surgery | 4 (6.2%) | 10 (11.2%) | | 0.291^¶^ | |  |
| Parenchyma firmness | | |  | | 0.097^¶^ | |
| Soft | 56 (87.5) | 68 (76.4) | |  | |  |
| Hard | 8 (12.5) | 21 (23.6) | |  | |  |
| Operative time (min) * | 200 (100~460) | 210 (110~440) | | 0.325^#^ | |  |
| Spleen preservation | 12 (18.8%) | 13 (14.6%) | | 0.494 | |  |
| Concomitant PV/SMV wall resection | 8 (12.5%) | 15 (16.9%) | | 0.457 | |  |
| Estimated blood loss (ml) * | 100 (20~1000) | 200 (20~1500) | | 0.076^#^ | |  |
| Transfusion | 6 (9.4%) | 17 (19.1%) | | 0.097 | |  |
| Duration of drainage (days) * | 10 (6~60) | 8 (3~60) | | 0.032^#^ | |  |
| Postoperative LOS (days) * | 9 (6~25) | 10 (6~26) | | 0.486^#^ | |  |
| Clavien-Dindo classification | | | | 1.000^¶^ | |  |
| Grade I or II | 46 (71.9%) | 42 (47.2%) | |  | |  |
| Grade IIIa† | 2 (3.1%) | 1 (1.1%) | |  | |  |
| Grade IIIb | 0 (0) | 0 (0) | |  | |  |
| Grade IV or V | 0 (0) | 0 (0) | |  | |  |
| 90-day mortality | 0 (0) | 0 (0) | | 1.000 | |  |

Values in parentheses are percentages unless indicated otherwise; *values are median (range). PV, portal vein. SMV, superior mesentery vein. LOS, length of stay. † Including one delayed gastric emptying and one peri-pancreatic fluid accumulation with fever needing reintervention. § χ2 test, except ¶ Fisher’s exact test and # Mann–Whitney U test.
